# Chasm in primary care provision in a universal health system: Findings from a nationally representative survey of health facilities in Malaysia

**DOI:** 10.1371/journal.pone.0172229

**Published:** 2017-02-14

**Authors:** Huy Ming Lim, Sheamini Sivasampu, Ee Ming Khoo, Kamaliah Mohamad Noh

**Affiliations:** 1 Healthcare Statistics Unit, National Clinical Research Centre, National Institutes of Health, Ministry of Health, Kuala Lumpur, Malaysia; 2 Department of Primary Care Medicine, Faculty of Medicine, University of Malaya, Kuala Lumpur, Malaysia; 3 Family Health Development Division, Ministry of Health, Putrajaya, Malaysia; Foundation for Medical Research, INDIA

## Abstract

**Background:**

Malaysia has achieved universal health coverage since 1980s through the expansion of direct public provision, particularly in rural areas. However, no systematic examination of the rural-urban distribution of primary care services and resources has been conducted to date for policy impact evaluation.

**Methods:**

We conducted a national cross-sectional survey of 316 public and 597 private primary care clinics, selected through proportionate stratified random sampling, from June 2011 through February 2012. Using a questionnaire developed based on the World Health Organization toolkits on monitoring health systems strengthening, we examined the availability of primary care services/resources and the associations between service/resource availability and clinic ownership, locality, and patient load. Data were weighted for all analyses to account for the complex survey design and produce unbiased national estimates.

**Results:**

Private primary care clinics and doctors outnumbered their public counterparts by factors of 5.6 and 3.9, respectively, but the private clinics were significantly less well-equipped with basic facilities and provided a more limited range of services. Per capita densities of primary care clinics and workforce were higher in urban areas (2.2 clinics and 15.1 providers per 10,000 population in urban areas versus 1.1 clinics and 11.7 providers per 10,000 population in rural areas). Within the public sector, the distribution of health services and resources was unequal and strongly favored the urban clinics. Regression analysis revealed that rural clinics had lower availability of services and resources after adjusting for ownership and patient load, but the associations were not significant except for workforce availability (adjusted odds ratio [OR]: 0.82; 95% confidence interval [CI]: 0.71–0.96).

**Conclusions:**

Targeted primary care expansion in rural areas could be an effective first step towards achieving universal health coverage, especially in countries with limited healthcare resources. Nonetheless, geographic expansion alone is inadequate to achieve effective coverage in a dichotomous primary care system, and the role of the private sector in primary care delivery should not be overlooked.

## Introduction

The concept of universal health coverage (UHC) has taken center stage in the global health agenda in recent years. Target 3.8 of the new Sustainable Development Goals (SDGs) is to “achieve UHC, including financial risk protection, access to quality essential healthcare services and access to safe, effective, quality and affordable essential medicines and vaccines for all” [1]. UHC holds great promise for poverty alleviation and health improvement for countries at all income levels [2–4]. More than 100 countries worldwide have embraced the UHC concept and are currently implementing health system reforms or other intervention programs with the aim of advancing the transition towards UHC [5].

Malaysia, an upper-middle-income country in the World Health Organization (WHO) Western Pacific region, has achieved UHC through the expansion of direct public provision and been hailed as a learning example for other countries in pursuing UHC [6]. Malaysia has been reported to have achieved UHC in 1980s when per capita income was $3,700 (in 1990 international dollars), compared to $9,700 for Japan and $8,700 for Sweden, demonstrating that UHC can be achieved at relatively low income levels [6]. Additionally, Malaysia has been able to achieve levels of health status comparable to high-income countries with relatively lower health expenditures, spending about 4–5% of gross domestic product (GDP) on health, compared to an average of 11.6% in high-income countries [7]. Such success has been attributed to the government’s targeted expansion of rural primary care and effective intervention programs in high priority areas, in particular maternal and child health [6,8].

Adopting a pro-poor approach, a public primary care delivery network consisting of health clinics, midwife clinics, community clinics, and mobile clinics was set up and expanded in phases in rural areas soon after Independence in 1957 in Malaysia to bring essential health services to the poorer rural communities, where household incomes were too low to support a private healthcare market [9]. Higher levels of care were available through a referral system. Medical doctors, community nurses, midwives, and assistant medical officers (equivalent to physician associates in the United Kingdom and physician assistants in the United States) formed the nucleus of the initial primary care workforce, which expanded over time to include an increasing number of medical doctors and allied health professionals [9]. As a result of this targeted expansion, the ratio of rural dwellers to health clinic dropped drastically from 638,000 rural dwellers per health center in 1960 to 21,697 rural dwellers per health center in 1986 [10]. Significant population health improvements took place over the same period. For instance, maternal mortality ratio plummeted from 282 per 100,000 live births in 1957 to 37 per 100,000 live births in 1985, an improvement unparalleled by most other developing countries [11].

While the rapid expansion of public provision in rural areas has led to significant health gains, the government’s focus on rural healthcare provision has also inadvertently contributed to the formation of a dichotomous primary care system. Public clinics which are funded through general tax revenues continue their roles as the main providers in rural areas, while urban populations are largely served by the private sector, for which out-of-pocket payments and private health insurance are the main financing mechanisms [9]. The high out-of-pocket payments in the private sector suggest that effective coverage is actually less than the 100% afforded by the tax-funded public system and could have significant implications for the sustainability of UHC in Malaysia.

Despite more than half a century of emphasis on expanding primary care in rural areas, no systematic examination of the rural-urban distribution of primary care services and workforce in both public and private sectors has been conducted to date for policy impact evaluation. Discussion of UHC has largely focused on the “financial protection for all” dimension [12,13], while the “access to essential services” dimension receives relatively little attention. Recent data from the National Health and Morbidity Survey (NHMS) has shown that rural-urban health inequalities continue to persist in Malaysia [14], highlighting possible gaps in health service provision and access to primary healthcare. Additionally, rapid urbanization and population redistribution through internal migration have transformed the Malaysian population from a predominantly rural population in 1970 (73% rural) to a predominantly urban population in 2010 (71% urban) [15]. The disproportionate rural-to-urban migration of young adults has also resulted in a rural population that has a higher proportion of older people and a lower proportion of young adults than the national averages [16]. These demographic transitions necessitate an assessment of the current service provision to enable future policy planning to meet the resultant changes in population needs.

To fill the critical data gaps and feed future policy design for health systems strengthening, we conducted a national survey of primary care clinics in Malaysia to examine the distribution of primary care services and workforce, with a particular focus on rural-urban distribution.

## Methods

### Study design and participants

We analyzed data from the National Healthcare Establishment and Workforce Statistics Survey (NHEWS), a national cross-sectional survey of primary care clinics which we undertook from June 2011 through February 2012. The participants were public and private primary care clinics offering general health services in Malaysia. Hospital outpatient departments, university-based primary care clinics, private specialist clinics, community clinics, and mobile service delivery points were excluded from the study.

We assembled a list of all public and private primary care clinics operating in December 2010 based on the information provided by the Ministry of Health Malaysia. After exclusion based on eligibility criteria, the list comprised 806 public and 4,529 private clinics. We applied proportionate stratified random sampling to create a sample of 316 public and 651 private clinics. The sample size calculations were based on the differential response rate and service availability between public and private clinics reported in the previous cycle of NHEWS [17]. The sampling was stratified by ownership (public/private) and geographical region (13 states and three federal territories). Of the 651 private clinics sampled, 54 were excluded because they had closed, shifted to another address, or were unreachable. A final sample of 316 public clinics and 597 private clinics was used.

The Medical Research and Ethics Committee of the Ministry of Health Malaysia reviewed and approved the study protocol (approval number NMRR-09-842-4718). Waiver of the requirement to obtain informed consent from doctors was granted by the committee because all collected information was stored in a password-protected network environment and analyses relied exclusively on de-identified data.

### Survey development

We developed the survey questionnaire based on the WHO toolkits on monitoring health systems strengthening [18], with guidance from an expert panel of Ministry of Health representatives, primary care experts, and family medicine specialists. We identified key services and resources essential for the delivery of comprehensive primary care in the Malaysian context and formulated questionnaire items accordingly. The questionnaire was pilot tested and further refined to ensure face and content validity.

The final questionnaire consisted of questions on clinic profile (such as year of establishment and number of patient attendances), available resources (such as facilities, equipment, and number and type of health professionals), and availability of essential health services. We prepared the questionnaire in paper and electronic (in the form of web application) formats to provide alternative modes of participation and increase participation rate of clinics.

### Survey administration

The questionnaire was mailed to all sampled clinics via secure courier service together with an endorsement letter from the State Health Director, a postage-paid return envelope, and username and password for the web application. Follow-up calls were made to confirm receipt of the research pack. Nonrespondents received reminders via telephone two weeks after the mailing to increase response rate.

The study had internal and external quality assurance mechanisms in place. Data received via paper submission was examined for completeness and consistency prior to data entry. Electronic questionnaire had a built-in data verification module installed to ensure minimal data entry errors from the participants’ side. A designated team of researchers monitored the quality of data entered on a real-time basis. Erroneous data and outliers were verified and corrected as necessary via verbal or written communication with the representative of the corresponding clinic. Additionally, data on health workforce were triangulated with other data sources such as the Health Information Management System reports, the Malaysian Medical Council doctor database, the National Specialist Register, and databases of professional organizations.

### Statistical analysis

All survey data were weighted to account for the complex survey design and produce unbiased national estimates. The national estimates were calculated by first constructing a base weight for each clinic/doctor on the basis of the inverse probability of selection. The base weights were then post-stratified by locality (rural/urban) according to the population control totals for each sampling stratum provided by our final list of primary care clinics to reduce nonresponse bias. The rural-urban stratification was based on the Department of Statistics Malaysia definitions, whereby urban areas referred to gazetted areas with their adjoining built-up areas which had a population of 10,000 or more inhabitants, while agglomerations of less than 10,000 inhabitants were classified as rural areas.

The primary indicators used in this study were the density of primary care clinics/workforce and the availability of primary care workforce, services, facilities, and equipment. Clinic/workforce densities were defined as numbers of clinic/workforce per 10,000 population and calculated using mid-2010 population estimates from the Department of Statistics Malaysia. Availability was expressed as the percentage of clinics in which each workforce/service/facility/device was available in 2010 and the corresponding 95% confidence interval (CI). We used Rao-Scott modified Chi-square test to assess the differences in the weighted proportions. For post-hoc pairwise comparisons, non-overlapping 95% CIs were used as an indicator of statistical significance.

We applied generalized linear models with a logarithmic link to examine the associations between service/resource availability and clinic ownership, locality, and patient load. Response variable was mean availability of services/resources, expressed as the proportion of tracer items within each domain which were present and functioning in each clinic (Table 1). The overall availability was the unweighted average of the domain scores. We calculated odds ratios by exponentiation of the regression coefficients and the corresponding 95% CIs by means of Taylor series linearization. Sensitivity analyses were performed for different subtypes of services to examine the possible differences in associations.

**Table 1:** Calculations of mean availability for regression analysis.

Domain	Tracer items	Mean availability
Health workforce*	Medical doctor Staff nurse (nurse with diploma/degree) Pharmacist Assistant medical officer	n/4, where n is the total number of workforce tracer items available in the clinic
Services	Acute illness management Chronic disease management Antenatal and postnatal care Low-risk deliveries Family planning Pap smear examination Clinical breast examination Medical examination Occupational health Smoking cessation Minor surgery Laboratory services Dispensary services	n/13, where n is the total number of service tracer items available in the clinic
Facilities	Triage system Ambulance Pharmacy Laboratory Autoclave Diagnostic/imaging	n/6, where n is the total number of facility tracer items available in the clinic
Devices	Peak flow meter Nebulizer Glucometer Doppler fetal monitor Nursing bag and set† Electrocardiogram machine Resuscitation trolley Defibrillator Full blood count machine Bilirubinometer Chemistry analyzer HbA1c analyzer Ultrasound machine X-ray machine	n/14, where n is the total number of device tracer items available in the clinic

HbA1c: glycated hemoglobin.

* Only registered health professionals with a diploma/degree in the respective fields were included in the analysis.

^†^ A bag containing necessary nursing equipment usually carried by nurses during home visits to allow them to carry out nursing procedures with ease.

We performed all statistical analyses using the R statistical software version 3.2.4 (R Foundation for Statistical Computing, Vienna, Austria) and the “survey” package [19]. A *P*-value of less than 0.05 was regarded as statistically significant.

## Results

Of the 913 eligible primary care clinics sampled, 549 completed the survey, yielding an overall response rate of 60.1% (55.2% weighted). The response rates for public and private clinics were 76.3% (76.1% weighted) and 51.6% (51.5% weighted), respectively. Characteristics of the clinics and the corresponding national estimates are summarized in Table 2. About two-thirds (64.4%) of public clinics were located in rural areas, while the vast majority (90.7%) of private clinics were located in urban areas. The median number of clinic attendances in public clinics was 133.8 (interquartile range [IQR]: 64.9–247.1) patients per day, more than four times higher than that recorded in private clinics (32.3 patients per day; IQR: 21.4–46.9 patients per day).

**Table 2:** Characteristics of participating primary care clinics and the corresponding national estimates.

Characteristic		Public			Private		
		No. of clinics (n = 241)	National estimate (N = 806)		No. of clinics (n = 308)	National estimate (N = 4,529)	
			***No***.	***% (95% CI)***		***No***.	***% (95% CI)***
Locality*							
	Rural	142	519	64.4	30	419	9.3
	Urban	99	287	35.6	278	4,110	90.7
Type of practice							
	Solo	NA	..	..	240	3,534	78.0 (73.5–82.6)
	Group	NA	..	..	68	995	22.0 (17.4–26.5)
Entrepreneurship							
	Sole proprietor	NA	..	..	242	3,584	79.1 (74.7–83.5)
	Corporate body	NA	..	..	25	331	7.3 (4.6–10.0)
	Partnership	NA	..	..	41	614	13.6 (9.8–17.3)
Operating hours†							
	Normal office hours only	43	132	16.4 (12.9–19.9)	NA	..	..
	Extended-hours‡	36	106	13.2 (10.0–16.4)	NA	..	..
	On-call services§	179	623	77.3 (73.7–80.9)	NA	..	..
	<24 hours	NA	..	..	296	4,354	96.1 (94.0–98.3)
	24 hours	NA	..	..	12	175	3.9 (1.7–6.0)
				***Median (IQR)***			***Median (IQR)***
Duration of establishment (years)		..	..	31 (19–41)	..	..	14 (7–24)
Patient attendances per day		..	..	133.8 (64.9–247.1)	..	..	32.3 (21.4–46.9)

CI: confidence interval; NA: not applicable; IQR: interquartile range.

* 95% CIs could not be calculated as estimates were post-stratified by rural-urban distribution within each stratum.

^†^ The sum is more than 100% because some public clinics offered both extended-hours and on-call services.

^‡^ Extended-hours services refer to regular clinic operation beyond the standard office hours.

^§^ On-call services are after-hour services where at least one healthcare provider could be called to help in cases of emergency.

### Clinic/Workforce density

Overall, there were 5,335 primary care clinics in Malaysia, corresponding to a clinic density of 1.9 clinics per 10,000 population (Table 3). Clinic density was twice higher in the urban areas than in rural areas (2.2 versus 1.1 clinics per 10,000 population, respectively). Of the 40,007 health professionals working in primary care clinics, 24,348 (60.9%) were certified health workforce (doctors, staff nurses [registered nurses with a diploma/degree in nursing], community nurses [registered nurses with a certificate in nursing], assistant medical officers, and pharmacists). Urban areas had higher densities of doctors and nurses, but the densities of certified support staff (staff nurses, community nurses, and assistant medical officers) were higher in rural areas.

**Table 3:** Number and distribution of primary care clinics and health workforce.

			Overall		Rural		Urban	
			No.	Density per 10,000 population*	No.	Density per 10,000 population*	No.	Density per 10,000 population*
Clinics			5,335	1.9	938	1.1	4,397	2.2
	Public		806	0.3	519	0.6	287	0.1
	Private		4,529	1.6	419	0.5	4,110	2.0
Health workforce			40,007	14.1	9,589	11.7	30,417	15.1
	Doctor		8,178	2.9	1,014	1.2	7,164	3.6
	Nursing staff†		28,311	10.0	6,984	8.5	21,326	10.6
		Staff nurses	5,223	1.8	2,093	2.5	3,130	1.6
		Community nurses	7,429	2.6	3,433	4.2	3,996	2.0
		Nursing aides	15,658	5.5	1,458	1.8	14,200	7.1
	Assistant medical officer‡		2,468	0.9	1,262	1.5	1,206	0.6
	Pharmacist		1,050	0.4	329	0.4	721	0.4
	Certified§		24,348	8.6	8,131	9.9	16,218	8.1

Number might not add up because of rounding.

* Malaysian population size in mid-2010: overall = 28,334,135, rural = 8,209,165, urban = 20,124,970.

^†^ Staff nurses refer to registered nurses with a degree or diploma in nursing. Community nurse are registered nurses with a certificate in nursing. Nursing aides are clinic assistants with little to no formal training.

^‡^ Equivalent to physician associate in the United Kingdom and physician assistant in the United States.

^§^ Certified health workforce includes doctors, staff nurses, community nurses, assistant medical officers, and pharmacists.

### Service delivery and capacity

An estimated 93.5 million (95% CI: 87.6–99.4 million) outpatient visits were made to primary care clinics in 2010, corresponding to an average of 3.3 (95% CI: 3.1–3.5) visits per person per year. Private urban clinics accounted for 54.9% of all visits (51.4 million visits; 95% CI: 46.9–55.8 million visits), followed by public urban clinics at 25.8% (24.1 million visits; 95% CI: 20.6–27.6 million visits), public rural clinics at 12.8% (11.9 million visits; 95% CI: 10.6–13.3 million visits), and private rural clinics at 6.5% (6.1 million visits; 95% CI: 5.1–7.0 million visits). The corresponding per capita annual outpatient visits to each type of clinics were 1.8 (95% CI:1.7–2.0), 0.9 (95% CI: 0.7–1.0), 0.4 (95% CI: 0.4–0.5), and 0.2 (95% CI: 0.2–0.2) visits per person per year, respectively.

Table 4 shows the service delivery profile of primary care clinics in Malaysia. Significant variations were observed among different types of clinics with respect to clinic workload and availability of services and resources. Public clinics in urban areas reported a median patient load of 289.8 (IQR: 181.6–491.2) patients per day, higher than those reported by other clinics (public rural: 86.3 [IQR: 38.2–143.6] patients per day; private rural: 36.9 [IQR: 26.9–50.9] patients per day; private urban: 31.4 [IQR: 21.2–46.0] patients per day).

**Table 4:** Clinic characteristics and availability of health workforce, facilities, services, and medical devices in primary care clinics.

			Overall			Public						Private						*P*-value*
						Rural			Urban			Rural			Urban			
			No. of clinics (n = 549)	National estimate (N = 5,335)		No. of clinics (n = 142)	National estimate (N = 519)		No. of clinics (n = 99)	National estimate (N = 287)		No. of clinics (n = 30)	National estimate (N = 419)		No. of clinics (n = 278)	National estimate (N = 4,110)		
**Clinic characteristic**					***Median (IQR)***			***Median (IQR)***			***Median (IQR)***			***Median (IQR)***			***Median (IQR)***	
Duration of establishment (years)			..	..	16.0 (8.0–26.0)	..	..	33.0 (22.3–43.0)	..	..	25.7 (9.0–37.1)	..	..	8.5 (4.0–15.0)	..	..	15.0 (8.0–25.0)	<0.0001
Patient attendances per day			..	..	33.4 (21.5–59.0)	..	..	86.3 (38.2–143.6)	..	..	289.8 (181.6–491.2)	..	..	36.9 (26.9–50.9)	..	..	31.4 (21.2–46.0)	<0.0001
**Availability**				***No***.	***% (95% CI)***		***No***.	***% (95% CI)***		***No***.	***% (95% CI)***		***No***.	***% (95% CI)***		***No***.	***% (95% CI)***	
Health workforce																		
	Medical doctors		488	5,076	95.1 (94.7–95.5)	87	278	53.5 (49.8–57.2)	93	269	93.8 (90.6–97.1)	30	419	100.0	278	4,110	100.0	<0.0001
	Nursing staff		547	5,304	99.4 (98.6–100.0)	142	519	100.0	99	287	100.0	30	419	100.0	276	4,079	99.2 (98.2–100.0)	0.4442
		Staff nurses	248	1,159	21.7 (18.8–24.6)	118	395	76.1 (70.9–81.3)	97	284	98.9 (97.6–100.0)	4	63	15.0 (4.9–25.1)	29	417	10.1 (6.6–13.7)	<0.0001
		Community nurses	244	856	16.0 (15.0–17.1)	142	519	100.0	99	287	100.0	1	17	4.1 (0.0–11.3)	2	33	0.8 (0.0–1.9)	<0.0001
		Nursing aides	299	4,393	97.0 (95.1–98.9)	NA	..	..	NA	..	..	29	402	95.9 (88.7–100.0)	270	3,991	97.1 (95.1–99.1)	..
	Assistant medical officers		245	869	16.3 (15.1–17.4)	142	519	100.0	99	287	100.0	0	0	0.0	4	63	1.5 (0.1–3.0)	<0.0001
	Pharmacists		168	529	9.9 (9.1–10.8)	73	232	44.7 (40.3–49.0)	92	268	93.4 (89.3–97.5)	0	0	0.0	3	29	0.7 (0.0–1.6)	<0.0001
Facilities																		
	Triage system		224	1,617	30.3 (26.1–34.5)	85	292	56.3 (49.3–63.3)	61	174	60.5 (52.8–68.1)	5	82	19.5 (5.8–33.2)	73	1,070	26.0 (20.9–31.2)	<0.0001
	Ambulance		171	535	10.0 (9.4–10.7)	103	340	65.4 (59.7–71.2)	68	196	68.2 (61.0–75.4)	0	0	0.0	0	0	0.0	<0.0001
	Pharmacy		493	4,727	88.6 (85.6–91.6)	118	392	75.6 (70.6–80.6)	99	287	100.0	29	410	97.8 (93.9–100.0)	247	3,638	88.5 (84.7–92.3)	<0.0001
	Laboratory		241	1,320	24.7 (21.2–28.2)	99	310	59.8 (57.7–61.9)	93	270	94.0 (90.9–97.0)	3	35	8.4 (0.2–16.6)	46	704	17.1 (12.7–21.6)	<0.0001
	Autoclave		356	3,329	62.4 (57.8–67.0)	92	313	60.4 (54.3–66.5)	72	213	74.3 (68.2–80.4)	24	323	77.0 (63.8–90.3)	168	2,479	60.3 (54.5–66.1)	0.0224
	Diagnostic/imaging		122	1,144	21.4 (17.6–25.3)	11	36	7.0 (3.7–10.2)	40	117	40.7 (32.6–48.7)	5	78	18.5 (7.0–30.0)	66	914	22.2 (17.5–27.0)	0.0002
Services																		
	Acute illness management		547	5,304	99.4 (98.6–100.0)	142	519	100.0	99	287	100.0	30	419	100.0	276	4,079	99.3 (98.2–100.0)	0.4458
	Chronic disease management		547	5,323	99.8 (99.4–100.0)	142	519	100.0	98	285	99.3 (99.3–99.3)	30	419	100.0	277	4,100	99.8 (99.3–100.0)	0.4568
	Antenatal and postnatal care		462	4,131	77.4 (73.5–81.4)	142	519	100.0	89	262	91.3 (88.2–94.3)	28	390	93.1 (84.7–100.0)	203	2,960	72.0 (67.0–77.1)	<0.0001
	Low-risk deliveries		111	403	7.5 (6.8–8.2)	90	339	65.3 (59.2–71.5)	21	63	22.1 (15.5–28.7)	0	0	0.0	0	0	0.0	<0.0001
	Family planning		467	4,197	78.7 (74.5–82.8)	142	519	100.0	89	262	91.3 (88.2–94.3)	24	324	77.4 (63.8–91.1)	212	3,091	75.2 (70.1–80.4)	<0.0001
	Pap smear examination		454	3,970	74.4 (70.2–78.6)	142	519	100.0	98	285	99.4 (98.5–100.0)	18	241	57.6 (43.7–71.5)	196	2,925	71.2 (65.9–76.4)	<0.0001
	Clinical breast examination		461	4,105	76.9 (72.8–81.1)	138	502	96.7 (93.9–99.6)	96	279	97.2 (94.7–99.8)	20	255	60.9 (47.6–74.3)	207	3,068	74.6 (69.5–79.8)	<0.0001
	Medical examination		474	4,928	92.4 (90.5–94.2)	91	313	60.4 (54.2–66.6)	86	249	86.7 (81.6–91.8)	28	389	92.9 (85.5–100.0)	269	3,976	96.7 (94.6–98.8)	<0.0001
	Occupational health		168	1,650	30.9 (26.6–35.3)	35	117	22.6 (17.1–28.1)	34	96	33.6 (26.4–40.8)	5	66	15.7 (6.4–25.0)	94	1,371	33.4 (27.9–38.9)	0.0022
	Smoking cessation		218	1,421	26.6 (22.8–30.5)	79	266	51.2 (44.7–57.7)	76	219	76.2 (69.7–82.7)	8	118	28.1 (14.4–41.9)	55	819	19.9 (15.2–24.7)	<0.0001
	Minor surgery		425	4,447	83.4 (80.1–86.7)	96	359	69.2 (63.3–75.1)	65	187	65.1 (57.6–72.6)	27	379	90.5 (82.4–98.6)	237	3,522	85.7 (81.6–89.8)	<0.0001
	Laboratory		457	4,446	83.3 (80.0–86.7)	103	325	62.6 (59.5–65.7)	93	270	94.0 (90.9–97.0)	27	377	90.1 (79.9–100.0)	234	3,474	84.5 (80.4–88.7)	<0.0001
		In-house	283	1,934	36.2 (32.1–40.4)	99	314	60.5 (57.0–64.0)	89	258	89.9 (85.7–94.0)	5	60	14.3 (3.5–25.1)	90	1,302	31.7 (26.4–37.0)	<0.0001
		Outsourced	341	3,975	74.5 (70.9–78.2)	46	145	28.0 (22.9–33.1)	45	129	44.9 (37.3–52.6)	26	368	87.9 (77.2–98.6)	224	3,333	81.1 (76.5–85.6)	<0.0001
	Dispensary		549	5,335	100.0	142	519	100.0	99	287	100.0	30	419	100.0	278	4,110	100.0	..
Devices																		
	Peak flow meter		398	3,091	57.9 (53.6–62.3)	136	496	95.5 (92.3–98.7)	97	283	98.6 (96.9–100.0)	15	210	50.2 (40.1–60.3)	150	2,102	51.1 (45.6–56.7)	<0.0001
	Nebulizer		525	5,008	93.9 (91.5–96.2)	142	519	100.0	98	285	99.1 (97.8–100.0)	29	399	95.3 (87.9–100.0)	256	3,806	92.6 (89.6–95.6)	0.0716
	Glucometer		531	5,136	96.3 (94.5–98.1)	139	505	97.4 (95.0–99.8)	97	282	98.2 (96.2–100.0)	30	419	100.0	265	3,930	95.6 (93.3–98.0)	0.1756
	Doppler fetal monitor		205	984	18.4 (15.7–21.2)	117	422	81.4 (75.6–87.1)	65	191	66.7 (59.5–73.8)	1	16	3.7 (0.0–9.6)	22	354	8.6 (5.2–12.1)	<0.0001
	Nursing bag and set†		341	2,392	44.8 (40.4–49.3)	137	501	96.5 (93.9–99.2)	92	270	93.9 (90.4–97.5)	12	169	40.4 (25.7–55.1)	100	1,453	35.3 (29.8–40.9)	<0.0001
	Electrocardiogram machine		451	4,418	82.8 (79.4–86.2)	98	310	59.7 (56.9–62.6)	92	266	92.8 (89.1–96.4)	24	343	81.8 (68.8–94.7)	237	3,499	85.1 (80.9–89.3)	<0.0001
	Resuscitation trolley		424	3,796	71.1 (66.9–75.4)	121	425	82.0 (76.3–87.6)	96	279	97.2 (94.6–99.8)	16	231	55.0 (40.9–69.2)	191	2,861	69.6 (64.3–74.9)	<0.0001
	Defibrillator		126	439	8.2 (6.9–9.6)	59	181	34.8 (30.0–39.6)	61	176	61.2 (53.7–68.7)	2	23	5.5 (0.0–12.7)	4	60	1.5 (0.0–2.9)	<0.0001
	Full blood count machine		187	709	13.3 (11.4–15.2)	83	262	50.6 (47.3–53.8)	92	266	92.6 (89.1–96.1)	2	25	5.9 (0.0–13.0)	10	156	3.8 (1.5–6.1)	<0.0001
	Bilirubinometer		183	679	12.7 (11.0–14.5)	88	274	52.7 (49.0–56.5)	83	244	84.9 (80.1–89.7)	0	0	0.0	12	161	3.9 (1.7–6.1)	<0.0001
	Chemistry analyzer		100	445	8.3 (6.5–10.1)	29	98	18.8 (14.3–23.3)	56	161	56.2 (48.4–64.0)	2	21	5.1 (0.0–10.6)	13	165	4.0 (1.9–6.2)	<0.0001
	HbA1c analyzer		109	411	7.7 (6.3–9.1)	43	142	27.3 (22.0–32.6)	58	167	58.3 (50.7–65.9)	1	17	4.1 (0.0–11.3)	7	85	2.1 (0.6–3.5)	<0.0001
	Ultrasound machine		344	2,981	55.9 (51.3–60.4)	86	270	52.1 (48.7–55.5)	83	242	84.4 (79.7–89.2)	23	322	77.0 (64.1–89.9)	152	2,145	52.2 (46.4–58.0)	<0.0001
	X-ray machine		112	889	16.7 (13.3–20.0)	14	45	8.7 (5.1–12.2)	45	127	44.3 (36.4–52.1)	3	45	10.7 (0.6–20.8)	50	672	16.4 (12.2–20.5)	<0.0001

Number might not add up because of rounding. HbA1c: glycated hemoglobin.

* The *P*-values were estimated using design-based Kruskal-Wallis test for continuous variables and Rao-Scott modified Chi-square test for weighted proportions.

^†^ A bag containing necessary nursing equipment usually carried by nurses during home visits to allow them to carry out nursing procedures with ease.

Skill-mix composition differed significantly between public and private clinics. Public clinics were staffed by doctors, certified nurses, assistant medical officers, and pharmacists, while private workforce comprised primarily doctors and noncertified nursing aides. Certified nurses were available in only 15.0% and 10.1% of private clinics in rural and urban areas, respectively. Within the public sector, significantly lower proportions of rural clinics were staffed with medical doctors (53.5% versus 93.8% in urban areas, with an average of one doctor per clinic in rural areas and four doctors per clinic in urban areas), staff nurses (76.1% versus 98.9%), and pharmacists (44.7% versus 93.4%).

With the exception of pharmacy, the availability of basic facilities in primary care clinics was lower than desired. Triage system, ambulance, laboratory, and diagnostic/imaging room were available in less than one-thirds of the primary care clinics. None of the private clinics had ambulance, and significantly lower proportions of private clinics in both rural and urban areas had triage system and laboratory compared to their public counterparts. Unequal distribution of supporting facilities between rural and urban areas was observed within the public sector, where the proportions of public clinics having functioning pharmacy, laboratory, autoclave facilities, and imaging room were significantly lower in the rural areas. Similar patterns were observed with regard to the availability of devices, where private clinics were less well-equipped than public clinics, and within the public sector devices were available in significantly higher proportions of urban clinics compared to rural clinics.

Primary curative services such as acute and chronic disease management and dispensary services were provided in nearly all primary care clinics. Other primary care services showed more diverse patterns of availability. Maternal care and cancer screening services were available in more than 90% of public clinics in both rural and urban areas, but provided in only 50% to 75% of private clinics. Slightly more than 60% of public clinics in rural areas offered medical examination and laboratory services, significantly lower than those reported for private clinics and public urban clinics (>80% for all). Smoking cessation service was provided by less than one-quarter of private clinics, compared to 51.2% in rural and 76.2% in urban public clinics.

### Doctor profile

On the basis of 993 doctors who were practicing in the clinics that responded, a total of 8,178 doctors were estimated to be working in primary care clinics in 2010. About 80% of these doctors were private practitioners. Female accounted for more than two-thirds of the doctor workforce in the public sector, while the reverse is true for private sector (72.6% versus 32.5%, respectively, *P*<0.0001). In general, doctors in the private sector were older compared to their public counterparts (median age: 49 [IQR: 41–59] years versus 33 [IQR: 28–39] years, *P*<0.0001) and had worked more years in the primary care setting (median years of experience: 17 [IQR: 10–26] years versus 3 [IQR: 1–8] years, *P*<0.0001). About 12% of public doctors were specialists, compared to only 6.4% in the private sector. Table 5 shows the comparisons between public and private doctors stratified by rural-urban classification.

**Table 5:** Profile of primary care doctors.

		Overall			Public						Private						*P*-value*
					Rural			Urban			Rural			Urban			
		No. of doctors (n = 993)	National estimate (N = 8,178)		No. of doctors (n = 153)	National estimate (N = 535)		No. of doctors (n = 398)	National estimate (N = 1,139)		No. of doctors (n = 34)	National estimate (N = 479)		No. of doctors (n = 408)	National estimate (N = 6,025)		
				***Median (IQR)***			***Median (IQR)***			***Median (IQR)***			***Median (IQR)***			***Median (IQR)***	
Age (years)		..	..	46.0 (38.0–56.0)	..	..	30.0 (28.0–36.1)	..	..	34.0 (29.0–40.4)	..	..	45.5 (40.0–51.0)	..	..	49.0 (42.0–59.0)	<0.0001
Primary care experience (years)		..	..	14.0 (6.0–24.0)	..	..	2.3 (1.0–6.6)	..	..	4.0 (1.4–8.0)	..	..	12.6 (7.0–20.7)	..	..	17.0 (10.0–26.6)	<0.0001
			***No***.	***% (95% CI)***		***No***.	***% (95% CI)***		***No***.	***% (95% CI)***		***No***.	***% (95% CI)***		***No***.	***% (95% CI)***	
Gender																	<0.0001
	Female	549	3,329	40.7 (37.0–44.4)	106	378	70.7 (64.5–76.9)	299	837	73.5 (69.7–77.3)	6	82	17.2 (7.3–27.2)	138	2,031	33.7 (28.8–38.6)	..
	Male	444	4,849	59.3 (55.6–63.0)	47	157	29.3 (23.1–35.5)	99	301	26.5 (22.7–30.3)	28	396	82.8 (72.8–92.7)	270	3,994	66.3 (61.4–71.2)	..
Qualification†																	0.0126
	Medical officer	903	7,558	92.4 (90.1–94.8)	132	462	86.3 (82.8–89.8)	354	1,007	88.4 (86.2–90.6)	34	479	100.0	383	5,611	93.1 (90.0–96.3)	..
	Specialist	90	619	7.6 (5.2–9.9)	21	73	13.7 (10.2–17.2)	44	132	11.6 (9.4–13.8)	0	0	0.0	25	414	6.9 (3.7–10.0)	..
Postgraduate qualification																	..
	Postgraduate qualifications in family medicine	76	416	67.2 (52.7–81.6)	20	68	93.2 (84.4–100.0)	42	126	95.5 (89.7–100.0)	NA	..	..	14	222	53.5 (31.2–75.9)	..
	Masters of Public Health	5	43	6.9 (0.0–14.5)	1	5	6.8 (0.0–15.6)	2	6	4.5 (0.0–10.3)	NA	..	..	2	32	7.7 (0.0–18.9)	..
	Others	9	160	25.9 (12.2–39.5)	0	0	0.0	0	0	0.0	NA	..	..	9	160	38.7 (17.8–59.7)	..

Number might not add up because of rounding.

* The *P*-values were estimated using design-based Kruskal-Wallis test for continuous variables and Rao-Scott modified Chi-square test for weighted proportions.

^†^ Medical officer refers to a registered medical practitioner without postgraduate qualification. Specialist refers to a registered medical practitioner with a postgraduate qualification recognized by both the Ministry of Health and the Public Service Department of Malaysia.

### Determinants of service/resource availability

The multivariate analyses showed that service/resource availability was strongly associated with clinic ownership and patient load (Table 6). In general, more services/resources were available in public clinics and clinics with higher patient loads. The association pattern was rather consistent across different domains. Rural clinics generally had lower availability of services and resources, but the associations were not significant except for workforce availability. Sensitivity analyses for the service domain revealed that rural clinics had higher availability of maternal health services but lower availability of occupational health services compared to clinics located in the urban areas (Table 7).

**Table 6:** Factors associated with service/resource availability.

		Workforce			Facility			Service			Device			Overall		
		Unadjusted OR (95% CI)	Adjusted OR (95% CI)	*P*-value	Unadjusted OR (95% CI)	Adjusted OR (95% CI)	*P*-value	Unadjusted OR (95% CI)	Adjusted OR (95% CI)	*P*-value	Unadjusted OR (95% CI)	Adjusted OR (95% CI)	*P*-value	Unadjusted OR (95% CI)	Adjusted OR (95% CI)	*P*-value
Intercept		..	1.85 (1.33–2.58)	0.0003	..	0.76 (0.55–1.06)	0.1050	..	2.41 (1.95–2.98)	<0.0001	..	0.99 (0.77–1.28)	0.9594	..	1.33 (1.08–1.64)	0.0074
Ownership																
	Public	1.00	1.00	..	1.00	1.00	..	1.00	1.00	..	1.00	1.00	..	1.00	1.00	..
	Private	0.11 (0.10–0.12)	0.20 (0.14–0.28)	<0.0001	0.36 (0.32–0.40)	0.64 (0.46–0.87)	0.0055	0.59 (0.53–0.66)	0.86 (0.72–1.03)	0.1090	0.28 (0.26–0.30)	0.50 (0.39–0.65)	<0.0001	0.29 (0.28–0.31)	0.50 (0.41–0.62)	<0.0001
Locality																
	Urban	1.00	1.00	..	1.00	1.00	..	1.00	1.00	..	1.00	1.00	..	1.00	1.00	..
	Rural	2.14 (1.98–2.31)	0.82 (0.71–0.96)	0.0121	1.40 (1.22–1.61)	0.95 (0.78–1.16)	0.6073	1.23 (1.08–1.39)	1.02 (0.87–1.19)	0.8047	1.55 (1.41–1.71)	0.93 (0.80–1.08)	0.3164	1.51 (1.40–1.64)	0.94 (0.83–1.05)	0.2790
Patient attendances per day																
	<30	1.00	1.00	..	1.00	1.00	..	1.00	1.00	..	1.00	1.00	..	1.00	1.00	..
	30–100	1.11 (1.01–1.23)	1.08 (0.97–1.19)	0.1488	1.29 (1.09–1.52)	1.28 (1.08–1.51)	0.0048	1.25 (1.08–1.45)	1.24 (1.07–1.45)	0.0046	1.39 (1.25–1.55)	1.38 (1.23–1.54)	<0.0001	1.23 (1.14–1.34)	1.22 (1.12–1.32)	<0.0001
	100–150	5.34 (3.00–9.48)	2.36 (1.57–3.55)	<0.0001	2.81 (1.99–3.96)	2.18 (1.54–3.10)	<0.0001	1.96 (1.51–2.54)	1.78 (1.32–2.40)	0.0002	3.37 (2.45–4.64)	2.31 (1.75–3.04)	<0.0001	3.01 (2.24–4.05)	2.06 (1.63–2.61)	<0.0001
	>150	26.97 (10.20–71.31)	7.73 (3.01–19.87)	<0.0001	5.45 (3.93–7.57)	3.74 (2.30–6.06)	<0.0001	2.59 (2.20–3.06)	2.27 (1.81–2.85)	<0.0001	7.93 (5.49–11.44)	4.47 (2.84–7.04)	<0.0001	6.73 (4.92–9.23)	3.80 (2.62–5.52)	<0.0001

OR: odds ratio.

**Table 7:** Results of sensitivity analyses for the service domain.

		Maternal health*		Preventive services†		Occupational Health		Minor surgery		Laboratory services	
		Adjusted OR (95% CI)	*P*-value	Adjusted OR (95% CI)	*P*-value	Adjusted OR (95% CI)	*P*-value	Adjusted OR (95% CI)	*P*-value	Adjusted OR (95% CI)	*P*-value
Intercept		2.09 (1.46–2.98)	0.0001	2.90 (1.99–4.24)	<0.0001	0.24 (0.10–0.53)	0.0005	1.14 (0.54–2.44)	0.7283	0.40 (0.16–1.00)	0.0514
Ownership											
	Public	1.00	..	1.00	..	1.00	..	1.00	..	1.00	..
	Private	0.22 (0.16–0.30)	<0.0001	0.62 (0.44–0.86)	0.0044	1.84 (0.88–3.86)	0.1060	4.37 (2.29–8.37)	<0.0001	12.10 (5.14–28.45)	<0.0001
Locality											
	Urban	1.00	..	1.00	..	1.00	..	1.00	..	1.00	..
	Rural	1.70 (1.43–2.03)	<0.0001	0.84 (0.62–1.15)	0.2738	0.54 (0.34–0.86)	0.0097	1.50 (0.82–2.75)	0.1890	1.41 (0.57–3.48)	0.4582
Patient attendances per day											
	<30	1.00	..	1.00	..	1.00	..	1.00	..	1.00	..
	30–100	1.46 (1.18–1.80)	0.0006	1.30 (1.02–1.66)	0.0348	1.23 (0.77–1.95)	0.3826	1.48 (0.83–2.63)	0.1833	1.24 (0.71–2.17)	0.4462
	100–150	1.33 (0.90–1.96)	0.1580	2.61 (1.49–4.56)	0.0008	1.53 (0.48–4.89)	0.4747	0.97 (0.50–1.88)	0.9246	17.22 (5.32–55.75)	<0.0001
	>150	0.72 (0.47–1.09)	0.1234	4.81 (3.15–7.35)	<0.0001	3.59 (1.52–8.52)	0.0038	1.81 (0.90–3.62)	0.0951	..	..

OR: odds ratio.

* Maternal health services include antenatal and postnatal care as well as low-risk deliveries.

^†^ Preventive services include medical examination, family planning, pap smear examination, clinical breast examination, and smoking cessation.

## Discussion

On the basis of a large, nationally representative sample of primary care clinics, our study indicates that inequalities exist in primary care provision in Malaysia, both between public and private sectors and between rural and urban areas. Private clinics and doctors practicing in private clinics outnumber their public counterparts by several-fold, but private clinics are significantly less well-equipped with basic facilities and devices and provide fewer primary care services, particularly maternal and preventive services. Public clinics have better skill-mix of primary care providers, but doctors in the private sector had more years of primary care experience. Per capita densities of primary care clinics and workforce are higher in urban areas. Within the public sector, two-thirds of the clinics are located in rural areas, but the availability of health services and resources is higher in the urban clinics.

The marked inequalities in primary care delivery could have negative implications for both rural and urban populations. Findings from the National Health and Morbidity Survey (NHMS) 2011 showed that rural populations have higher burden of undiagnosed chronic conditions and malnutrition [14]. The high morbidity likely reflects unmet healthcare needs among rural populations, in addition to the well-documented differentials in social determinants of health. Additionally, since private clinics provide a more limited range of services and rely largely on out-of-pocket payments for funding, the unabated expansion of private clinics in recent years could undermine access to essential and quality health services. This is supported by another finding from NHMS 2011, where recent users of private facilities reported a lower willingness to recommend outpatient health facilities used compared to users of public facilities, implying a lower level of satisfaction with the quality of care received among private patients [14]. Policies to improve the comprehensiveness of services provided in both public and private clinics, taking into account the needs of the local population, is of grave importance to improve the health of the nation. Additionally, the dichotomous public-private divide and fragmented care provision also highlight the urgent need for uniform standards to integrate public and private primary care services to ensure a more responsive and sustainable primary care system.

Our study also reveals concerning inadequacies in primary care service capacity, which could limit rapid scale-up and expansion of primary care services in the near future. Malaysia had 2.9 primary care doctors per 10,000 population in 2010, falling short of the benchmarks of 7.1–11.0 general practitioners per 10,000 population recommended by the Australian authorities for optimal primary care provision [20]. The density would be even lower if only doctors with postgraduate qualifications are considered, as they account for only 7.6% of the primary care doctor workforce in Malaysia. In comparison, Australia had 11.2 full-time equivalent general practitioners per 10,000 population in 2012 [21], while the United Kingdom reported a density of 6.0 general practitioners per 10,000 population in 2013 [22]. The low doctor density is further compounded by the maldistribution of doctors, with the density being three times lower in rural areas. These findings suggest that Malaysia might benefit from targeted area-level policies aiming at increasing the supply of primary care doctors and, at the same time, reducing geographical inequalities in access. Studies have shown that increased supply of primary care doctors is associated with better population health for various health outcomes, but the mere increase in doctor supply will not necessarily lead to a more equal distribution [23–25]. In addition, noncertified nursing aides who are employed in the private sector and constitute the bulk (39.1%) of primary care workforce in Malaysia represent an area of high potential for improvement. The finding, while alarming, suggests that in-service training of this existing workforce might serve as a cost-effective and time-efficient strategy to increase the quality of primary care in Malaysia.

Our regression analyses indicate strong associations between service/resource availability and clinic ownership as well as patient load. Private clinics are significantly less likely to have the full range of essential services and resources available. The association between patient load and availability of services and resources exists in gradations, where clinics with higher patient volumes also have more health workforce, facilities, and services available. After adjusting for ownership and patient load, rural clinics generally have lower availability of services and resources, but the associations are not significant except for workforce availability. These findings argue for the success of the Malaysian government’s long-term demand-driven strategy for health system strengthening, where clinics with higher patient loads are allocated with more resources in order to achieve operational efficiencies and reach more patients with the limited resources.

However, the possibility of an inverse relationship between patient load and service availability could not be ruled out. Results from other countries have shown that the availability of services, facilities, and workforce could affect people’s likelihood to utilize services, where people are likely to bypass the nearest primary care facilities in favor of more distant facilities when services and resources at local facilities are limited [26,27]. The low clinic attendances in rural clinics might reflect the population reluctance to use the services rather than a true intrinsic lower health needs in the rural populations. This is substantiated by findings from previous studies, which showed that the prevalence of recent utilization of outpatient care did not differ between rural and urban areas and that rural residents were more likely to bypass the nearest health facilities, with perceived poor quality of care being the most frequently cited reason [14,28]. Moreover, in the presence of similar or higher disease burdens, lower clinic densities in the rural areas should result in higher patient load in the rural clinics, but the opposite finding is observed. The possibility is there that patients from rural areas visit clinics in urban areas in order to access a more comprehensive range of services. This, coupled with the disproportionate presentations of urban patients to the public clinics for more comprehensive and affordable services, leads to the congestion in the public urban clinics. Further research is needed to explore these and other possible explanations for these findings.

Our study shows that healthcare inequalities can persist even after achieving UHC for decades. This concurs with findings from the United Kingdom, which show that healthcare inequalities continue to exist despite the provision of UHC since 1948 through the National Health Service (NHS) [29,30]. Together, these findings show that the attainment of UHC is not the culmination of health systems strengthening. Sustained efforts to improve the accessibility and quality of care are necessary to reduce health and health system inequalities and improve population health, especially in the face of rising healthcare costs, demographic and epidemiological transitions, and rising expectations. The success story of Malaysia in achieving UHC suggests that targeted and demand-driven expansion in primary care provision could be an effective first step towards achieving UHC, especially in countries with limited healthcare resources. Nonetheless, the lower service availability in rural areas and the existence of a dichotomous primary care system serve as important reminders that geographic expansion alone is inadequate to achieve effective coverage and that partnerships between government and the private sector are vital to the achievement and sustenance of UHC. These insights are particularly relevant to countries currently in the process of developing and instituting strategies to achieve UHC, as studies have shown that early policy decisions could affect the efficiency and sustainability of UHC system and are often difficult to change once institutionalized [31].

Our study has several limitations. First, our study was cross-sectional in nature, and no temporal or causal relationships could therefore be determined. Second, we measured only the availability of different health services and resources and did not capture the quality of care nor patient satisfaction with the services provided. While the mere availability of infrastructures does not guarantee service quality and patient satisfaction, data on availability as a pre-condition for quality could provide a basis for future analysis and planning. Assessments of service quality and patient satisfaction would be the logical next step in primary care monitoring. Third, although we developed our questionnaire on the basis of WHO toolkits on monitoring health systems strengthening, significant changes were made to the construct of different domains, rendering cross-country comparisons difficult. The changes were necessary because the WHO toolkits focus on low and lower-middle-income countries and include many indicators which we found unsuitable to be applied in the Malaysian context. Finally, the subgroup of private rural clinics had a relatively small sample size (n = 30), which resulted in relatively wide confidence intervals and national estimates that should be interpreted with caution.

Being the first national survey to comprehensively examine the distribution of primary care services and resources in Malaysia, our study contributes to the primary care strengthening in Malaysia by providing benchmark information about the availability of services, facilities, and health workforce as of 2010. Our results serve to underscore the existence of health system inequalities in primary care provision in Malaysia and highlight the urgency to integrate the public and private provision of primary care. Persistent rural-urban inequalities in health and healthcare after half a century of focusing on rural health provision call for new national focus on improving the quality of care and readdressing the needs of population instead of mere geographic expansion. Further studies using quality-of-care indicators will be necessary to inform future strengthening of primary care.

## Supporting information

S1 Appendix Survey instrument (paper format). (PDF)

S2 Appendix De-identified survey data: Primary care clinic profile. (XLSX)

S3 Appendix De-identified survey data: Primary care doctor profile. (XLSX)
