# Supplementary material for: Chasm in primary care provision in a universal health system: Findings from a nationally representative survey of health facilities in Malaysia
Source: PLoS One. 2017 Feb 14;12(2):e0172229. doi: 10.1371/journal.pone.0172229 (PMC5308860; doi:10.1371/journal.pone.0172229)
Supplement: S1 Appendix — (PDF) [file pone.0172229.s001.pdf]

NATIONAL HEALTHCARE ESTABLISHMENT AND WORKFORCE SURVEY (NHEWS) FORM

PRIMARY CARE

A Collaboration of:

Medical Development Division, Medical Practice Division, Family Health Development Division, Medical Device Bureau, and Clinical Research Centre, National Institutes of Health.

Copyright © National Healthcare Statistics Initiative. All rights reserved.

INSTRUCTIONS TO PRIMARY CARE

1. Section 1 is best filled by the Person in-Charge in the Administration Department.
2. Section 2 to Section 6 is best filled by Heads of Department or Person in-Charge of the relevant services/ departments.
3. Any queries please contact the Healthcare Statistics Unit, Clinical Research Centre at 03-4043 9300/ 9400 or fax 03-4043 9500 or email hsu@crc.gov.my.

National Healthcare Establishment and Workforce Survey (NHEWS): Primary Care

General Instructions

1. Please ensure that all sections of the NHEWS: Primary Care questionnaire have been provided. These include:
    - SECTION 1: ESTABLISHMENT DETAILS
    - SECTION 2: FACILITIES (2010)
    - SECTION 3: HEALTHCARE ACTIVITIES & SERVICES (2010)
    - SECTION 4: HEALTH WORKFORCE (2010)
    - SECTION 5: DOCTORS' DETAILS AS REPORTED ON 31/12/2010
    - SECTION 6: MEDICAL DEVICES (2010)

    If any section has not been provided in the manual please contact us.
2. Data collected is for the year 2010.
3. Registration and data submission may be conducted in two ways:
    - Option 1: NHEWS: Primary Care web (online) survey
    - Option 2: NHEWS: Primary Care paper survey
4. Please provide the contact information in the **Information of person coordinating this survey (Primary Care Survey, Section 1).**
    (This information will be used to contact your establishment for the purpose of data clarification or dissemination of project updates.)
5. Where check boxes are provided, you may check one or more boxes. Where radio buttons are provided, check one box only.
6. Where a particular variable is not available or not applicable at your establishment, please enter 0.

Option 1 - NHEWS: Primary Care web(online) survey

1. Unique usernames will be provided.
    (Each clinic will have a unique username and password that will enable viewing and editing of data for that respective clinic. To ensure the security of your data, please limit the number of users.)
2. Log on to our website http://www.crc.gov.my/nhsi
3. After completing each section, please click the "Save" button.

Option 2 - NHEWS: Primary Care paper survey

1. The questionnaire has been provided in the manual and is downloadable from our website at http://www.crc.gov.my/nhsi.
2. All completed data must be returned using the pre-paid envelopes to the address below:

Kementerian Kesihatan Malaysia
Clinical Research Centre Surveys and Research Projects
3rd Floor, MMA House
124, Jalan Pahang
53000 Kuala Lumpur

Section 5: Doctors' Details as Reported on 31/12/2010

1. For establishments with only a few personnel please complete Section 5: Doctors' Details as Reported on 31/12/2010.
2. For establishments with a large number of staff kindly email the staff listing in an EXCEL spreadsheet or other electronic document to hsu@crc.gov.my or login to our website http://www.crc.gov.my/nhsi and upload using your username.
3. Doctor(s) to be included are Medical Officers (excluding House Officers), Specialists and Consultants.

Copyright © National Healthcare Statistics Initiative. All rights reserved.

NATIONAL HEALTHCARE ESTABLISHMENT AND WORKFORCE SURVEY (NHEWS) 2010 — PRIMARY CARE

Instruction: i) Where check boxes ☐ are provided, check (√) one or more boxes. Where radio buttons ◯ are provided, check (√) one box only.
ii) All data is for the year 2010 unless indicated otherwise.

Important:

1. If you prefer to complete the survey online, please go to www.crc.gov.my/nhsi
   Sekiranya anda ingin mengisi borang secara online, sila layari www.crc.gov.my/nhsi
2. If you are submitting a hard copy form,please return the form via fax to 03-4043 9500 or post using the prepaid envelope provided to the address below:-
   Sekiranya anda mengisi borang kertas, sila kembalikan melalui faks 03-4043 9500 atau melalui pos berdaftar ke alamat berikut:

   KEMENTERIAN KESIHATAN MALAYSIA,
   Clinical Research Centre Surveys and Research Projects,
   3rd Floor, MMA House, 124, Jalan Pahang, 53000 Kuala Lumpur.

3. For any enquiries, kindly contact the Healthcare Statistics Unit at 03-4043 9300, or fax 03-4043 9500, or email hsu@crc.gov.my
   Jika terdapat sebarang pertanyaan, sila hubungi Unit Statistik Perkhidmatan Kesihatan di talian: 03-4043 9300, atau faks 03-4043 9500, atau emel hsu@crc.gov.my

SECTION 1 : ESTABLISHMENT DETAILS / *MAKLUMAT PREMIS YANG DITUBUHKAN*

1. Name of establishment
* *Nama pertubuhan*

2. Owner of the establishment
Pemilik pertubuhan

3. District Health Office
* *Pejabat Kesihatan Daerah / bahagian / kawasan*
(For public clinics only/ *Klinik kerajaan sahaja)*

4. Address
* *Alamat*

Postcode *Poskod* ☐☐☐☐☐ **City / Town** *Bandar/Pekan*

District *Daerah*

State *Negeri*

◯ Perlis ◯ Selangor ◯ Pahang ◯ Sarawak
◯ Kedah ◯ Negeri Sembilan ◯ Terengganu ◯ Wilayah Persekutuan Kuala Lumpur
◯ Pulau Pinang ◯ Melaka ◯ Kelantan ◯ Wilayah Persekutuan Putrajaya
◯ Perak ◯ Johor ◯ Sabah ◯ Wilayah Persekutuan Labuan

5. Contact information
Talian

***Office phone** *Telefon pejabat* ☐☐☐ - ☐☐☐☐☐☐☐☐ **Fax** *Faks* ☐☐☐ - ☐☐☐☐☐☐☐☐

6. Entrepreneurship
* *Keusahawanan*
(For private clinics only *Klinik swasta sahaja)*

◯ Sole proprietor / *Tuan punya tunggal* ◯ Partnership / *Perkongsian*
◯ Corporate body / *Pertubuhan perbadanan* ◯ Society / *Persatuan*

7. Sector
* *Sektor*

◯ Ministry Of Health / *Kementerian Kesihatan* ◯ Armed forces / *Angkatan tentera*
◯ University / *Universiti* ◯ Non-government organization / *Organisasi bukan kerajaan*
◯ Private / *Swasta*

8. Year commenced operations
* *Tahun mula beroperasi* ☐☐☐☐

9. Type of practice
* *Jenis amalan*
(For private clinics only *Klinik swasta sahaja)*

◯ Individual / *Perseorangan* ◯ Group / *Berkumpulan*

10. Type of establishment
* *Jenis pertubuhan*
(For private clinics only *Klinik swasta sahaja)*

◯ General practice clinic / *Klinik perubatan am*
◯ Specialist clinic / *Klinik pakar* → Please specify / *Sila nyatakan*

11. Type of clinic
Jenis klinik
(For public clinics only *Klinik kerajaan sahaja)*

◯ Klinik kesihatan → ◯ Type 1 / *Jenis 1* ◯ Type 2 / *Jenis 2* ◯ Type 3 / *Jenis 3* ◯ Type 4 / *Jenis 4* ◯ Type 5 / *Jenis 5* ◯ Type 6 / *Jenis 6*
◯ Others, specify / *Lain-lain, sila nyatakan* →

12. Services provided
* *Perkhidmatan yang disediakan*
(For public clinics only *Klinik kerajaan sahaja)*

☐ Maternal Child Health (MCH) / *Kesihatan ibu dan anak* ☐ Dental / *Pergigian*
☐ Outpatient / *Perkhidmatan pesakit luar* ☐ Accident & Emergency services / *Perkhidmatan kecemasan dan trauma (kemalangan)*

13. Information of person coordinating this survey
Maklumat pihak bertanggungjawab bagi kaji selidik ini

***Name** *Nama*
***Designation** *Jawatan*
***Office phone** *Telefon pejabat* ☐☐☐ - ☐☐☐☐☐☐☐☐ Ext: ☐☐☐☐
Handphone *Telefon bimbit* ☐☐☐ - ☐☐☐☐☐☐☐☐
Fax *Faks* ☐☐☐ - ☐☐☐☐☐☐☐☐ **Email** *E-mel*

Copyright © National Healthcare Statistics Initiative. All rights reserved.

Instruction: i) Where check boxes ☐ are provided, check (√) one or more boxes. Where radio buttons ○ are provided, check (√) one box only.

SECTION 2 : FACILITIES / *KEMUDAHAN* (2010)

Note: Please tick the facilities that are available at your establishment. / *Sila tandakan kemudahan yang sedia ada di pertubuhan anda.*

1. Does your establishment have
Adakah pertubuhan anda memiliki

- ☐ A triage system for assessing patients / *Sistem triaj untuk saringan pesakit*
- ☐ Pharmacy space / *Ruang farmasi*
- ☐ Laboratory space / *Ruang makmal*
- ☐ Diagnostic/Imaging room / *Bilik diagnostik / imbasan*
- ☐ Ambulance services managed by your establishment / *Perkhidmatan ambulans yang diurus sendiri*
  - Number of functional ambulances available / *Bilangan ambulans yang berfungsi* ☐☐
- ☐ Autoclaving facilities for sterilisation of medical equipment / *Kemudahan autoklaf bagi pensterilan peralatan perubatan*
- ☐ None / *Tiada*

SECTION 3 : HEALTHCARE ACTIVITIES & SERVICES / *AKTIVITI & PERKHIDMATAN KESIHATAN* (2010)

1. Operating hours / *Waktu beroperasi*

a. Public clinics / *Klinik kerajaan*
- ☐ Office hours / *Waktu pejabat*
- ☐ Extended hours / *Lebih masa*
- ☐ After hours on call services / *Perkhidmatan atas panggilan*
- ☐ 10am – 10pm daily / *10 pagi - 10 malam setiap hari*

b. Private clinics / *Klinik swasta*
- ○ Less than 24 hours / *Kurang daripada 24 jam*
- ○ 24 hours / *24 jam*

2. Does your establishment have these services
Adakah pertubuhan anda menyediakan perkhidmatan

- ☐ Management of acute illnesses / *Rawatan penyakit akut*
- ☐ Management of chronic diseases / *Rawatan penyakit kronik*
- ☐ Antenatal & postnatal care / *Penjagaan antenatal & postnatal*
- ☐ Low risk deliveries / *Kelahiran berisiko rendah* → Total number / *Jumlah* ☐☐
- ☐ Family planning / *Perancangan keluarga*
- ☐ Pap smear (cervical screening) / *Pap smear*
- ☐ Clinical breast examinations / *Pemeriksaan klinikal payudara*
- ☐ Medical check-ups / examinations / *Pemeriksaan kesihatan*
- ☐ Occupational health / *Kesihatan cara kerja*
- ☐ Smoking cessation programmes / *Program berhenti merokok*
- ☐ Minor surgeries / *Pembedahan minor*
- ☐ Laboratory services / *Perkhidmatan makmal* → ☐ In-house / *Dalaman* ☐ Out-sourced / *Khidmat luar*
- ☐ Dispensing / *Pendispensan ubat*
- ☐ None / *Tiada*

**3. Total number of attendances** * **(With or without doctors' consultation)** *Jumlah kehadiran pesakit (dengan atau tanpa rundingan doktor)*	☐☐☐☐☐☐
**3.1 Face to face consults** *Perundingan secara bersemuka*	☐☐☐☐☐☐
**3.2 Home visits** *Lawatan ke rumah*	☐☐☐☐
**3.3 Total number of antenatal attendances** *Jumlah kehadiran pesakit antenatal*	☐☐☐☐
**3.4 Total number of children attendances** *Jumlah kehadiran pesakit kanak kanak*	☐☐☐☐

SECTION 4 : HEALTH WORKFORCE / *TENAGA KERJA BAGI PERKHIDMATAN KESIHATAN* (2010)

**1. Total number of medical doctors** * *Jumlah pegawai perubatan*	☐☐☐☐
**2. Total number of nursing staff** *Jumlah anggota kejururawatan*	
**2.1. Staff nurses** *Jururawat*	☐☐☐☐
**2.2. Community nurses** *Jururawat masyarakat*	☐☐☐☐
**2.3. Nursing aides** *Pembantu klinik*	☐☐☐☐
**3. Total number of pharmacists** *Jumlah pegawai farmasi*	☐☐☐☐
**4. Total number of assistant medical officers** *Jumlah penolong pegawai perubatan*	☐☐☐☐

Copyright © National Healthcare Statistics Initiative. All rights reserved.

Instruction: i) Where check boxes ☐ are provided, check (√) one or more boxes. Where radio buttons ○ are provided, check (√) one box only.

SECTION 5 : DOCTORS' DETAILS AS REPORTED ON 31/12/2010 / *MAKLUMAT TENAGA KERJA PERUBATAN PADA 31/12/2010*

For establishments with a large number of staff, kindly email the staff listing in an Excel spreadsheet using the format provided, to hsu@crc.gov.my
Bagi Institusi dengan bilangan kakitangan yang besar, sila hantar senarai kakitangan dalam excel dengan mengikuti format yang disediakan kepada hsu@crc.gov.my

For public clinics, please provide details of permanent / resident doctors only
Bagi Klinik kerajaan , sila senaraikan maklumat doktor tetap sahaja

**Title**<br>*Gelaran*	*** Name of doctor(s)**<br>(Name as per MyKad)<br>(Medical officers, specialists and consultants only)<br>*Nama pengamal perubatan*<br>*(Nama seperti yang tercetak pada MyKad)*<br>*(Pegawai perubatan, pakar perubatan dan pakar perunding sahaja)*	**Identification**<br>(Please fill in one or more of the information below)<br>*Pengenalan*<br>*(Sila isi satu atau lebih daripada yang berikut)*	**Type**<br>*Jenis*	**Qualification**<br>*Kelayakan*	**Post-graduate qualification**<br>*Kelayakan pascaijazah*	**Duration spent in primary care**<br>*Tempoh bertugas dalam bidang kesihatan primer*
		* MMC Number / *Nombor MMC* ☐☐☐☐☐<br>MyKad ☐☐☐☐☐☐ - ☐☐ - ☐☐☐☐<br>Other ID document No / *Nombor pengenalan yang lain* ____<br>Specify document type (if others) / *Sila nyatakan jenis dokumen*<br>○ Passport / *Pasport* ○ Others / *Lain- lain*<br>○ Armed Force ID / *Nombor Tentera atau Polis*	○ Permanent / resident *Tetap*<br>○ Visiting *Pelawat*	○ Specialist *Pakar* →<br>○ Medical officer *Pegawai perubatan*	○ Masters in Family Medicine UM / UKM / USM<br>○ FRACGP holder<br>○ Masters in Public Health<br>○ Others, specify: ____	☐☐ Years *Tahun*<br>☐☐ Months *Bulan*<br>ADD
		* MMC Number / *Nombor MMC* ☐☐☐☐☐<br>MyKad ☐☐☐☐☐☐ - ☐☐ - ☐☐☐☐<br>Other ID document No / *Nombor pengenalan yang lain* ____<br>Specify document type (if others) / *Sila nyatakan jenis dokumen*<br>○ Passport / *Pasport* ○ Others / *Lain- lain*<br>○ Armed Force ID / *Nombor Tentera atau Polis*	○ Permanent / resident *Tetap*<br>○ Visiting *Pelawat*	○ Specialist *Pakar* →<br>○ Medical officer *Pegawai perubatan*	○ Masters in Family Medicine UM / UKM / USM<br>○ FRACGP holder<br>○ Masters in Public Health<br>○ Others, specify: ____	☐☐ Years *Tahun*<br>☐☐ Months *Bulan*<br>ADD
		* MMC Number / *Nombor MMC* ☐☐☐☐☐<br>MyKad ☐☐☐☐☐☐ - ☐☐ - ☐☐☐☐<br>Other ID document No / *Nombor pengenalan yang lain* ____<br>Specify document type (if others) / *Sila nyatakan jenis dokumen*<br>○ Passport / *Pasport* ○ Others / *Lain- lain*<br>○ Armed Force ID / *Nombor Tentera atau Polis*	○ Permanent / resident *Tetap*<br>○ Visiting *Pelawat*	○ Specialist *Pakar* →<br>○ Medical officer *Pegawai perubatan*	○ Masters in Family Medicine UM / UKM / USM<br>○ FRACGP holder<br>○ Masters in Public Health<br>○ Others, specify: ____	☐☐ Years *Tahun*<br>☐☐ Months *Bulan*<br>ADD

Copyright © National Healthcare Statistics Initiative. All rights reserved.

Instruction: i) Where check boxes ☐ are provided, check (√) one or more boxes. Where radio buttons ○ are provided, check (√) one box only.

SECTION 6 : MEDICAL DEVICES / *PERALATAN PERUBATAN* (2010)

**Medical devices** *Peralatan perubatan*	**Number of equipment available in 2010** *Kuantiti peralatan pada 2010*	
	**Functioning** *Berfungsi*	**Non functioning** *Tidak berfungsi*
1. Peak flow meter		
2. Nebuliser		
3. Glucometer		
4. Doppler Fetal Monitor		
5. Nursing bag & set		
6. Electrocardiogram machine (ECG)		
7. Resuscitation (emergency) trolley equipped with resuscitating equipment and drugs		
8. Defibrillator		
9. Full blood count machine		
10. Bilirubinometer (Total bilirubin)		
11. Chemistry analyser		
12. HbA1C analyser		
13. Utrasound machine		
14. General radiology unit (X-ray machine)		

Copyright © National Healthcare Statistics Initiative. All rights reserved.

SECTION 4: HEALTH WORKFORCE (2010)

NO	DATA ELEMENT	DEFINITION
1	Medical doctors	Any medical practitioner registered with the Malaysian Medical Council
2	Staff nurses	Any registered nurse with a degree or diploma in nursing
3	Community nurses	Any registered nurse with a certificate in nursing
4	Nursing aides	Clinic assistants with little or no formal training
5	Assistant medical officers	An assistant medical officer registered with the Medical Assistant Board

SECTION 5: DOCTORS' DETAILS AS REPORTED ON 31/12/2010

NO	DATA ELEMENT	DEFINITION
1	Resident	An in-house medical practitioner who is available to diagnose and treat patients at your centre; and is a permanent staff in your establishment.
2	Visiting	A medical practitioner who is available to diagnose and treat patients at your centre but he/she is not a permanent staff at that centre. The medical practitioner may make regular visits (eg monthly/ biweekly/ weekly etc) to see patients or is accessible on an ad-hoc or emergency basis only.
3	Specialist	A registered medical practitioner with a post-graduate qualification recognized by both the Ministry of Health and the Public Service Department of Malaysia.

SECTION 6 : MEDICAL DEVICES 2010

NO	DATA ELEMENT	DEFINITION
1	Peak flow meter	A device used to measure air flow out of the lungs called the peak expiratory flow rate (PEFR), as a person forcefully blows into the device
2	Nebuliser	A device used to reduce a liquid medication to extremely fine cloudlike particles; useful in delivering medication to deeper parts of the respiratory tract
3	Glucometer	A battery-operated point-of-care device for determining the approximate blood glucose concentration
4	Electrocardiogram machine (ECG)	An electrical device intended for studying the activity of the heart based on changes in the voltage between pairs of electrodes placed at certain points on the skin
5	Resuscitation (emergency) trolley equipped with resuscitating equipment and drugs	A portable trolley containing the necessary equipment and drugs required in cardiopulmonary resuscitation and emergency care of patients
6	Defibrillator	A medical electrical device intended to defibrillate the heart by electrical pulses via electrodes when the heart's own conduction slows dangerously or stops. It may or may not have transcutaneous pacing capabilities of electrically stimulating the heart externally.
7	Full blood count machine	A machine that analyses the number and types of cells in the blood to generate the commonly used test known as the full blood count (FBC)/ complete blood count (CBC)
8	Bilirubinometer (Total bilirubin)	A device to measure the amount of bilirubin in the blood. The bilirubinometer is designed primarily to determine serum bilirubin levels in infants
9	Chemistry analyser	Laboratory analyzers that determine analyte concentrations in samples of plasma, serum, or less frequently in whole blood; they include analyzers used for blood gas and pH determinations.
10	HbA1C analyser	Medical equipment used to measure levels of HbA1c found in red blood cells (RBCs)
11	Doppler Fetal Monitor	Hand-held ultrasound transducer used to detect the heart beat of a foetus.
12	Nursing bag & set	A bag containing necessary articles usually carried by nurses during home visits to allow them to carry out nursing procedures with ease
13	Ultrasound machine	Imaging equipment, which uses high-frequency sound waves to create images of internal structures.
14	General radiology unit (X-ray machine)	A device used to acquire an x-ray image to visualise structures within the body

Copyright © National Healthcare Statistics Initiative. All rights reserved.

SECTION 1: ESTABLISHMENT DETAILS

NO	DATA ELEMENT	DEFINITION
1	Name of establishment	Name of establishment as per approved or licensed by the Ministry of Health Malaysia (Medical Practice Division-CKAPS)
2	Owner of the establishment	Name of individual/ organisation/ society that the establishment belongs to.
3	Address	Location where the establishment operates as per approved or licensed by the Ministry of Health Malaysia (Medical Practice Division- CKAPS)
4	Sole proprietor	A business entity owned by one person. The owner may operate on his or her own or may employ others. The owner of the business has total and unlimited personal liability of the debts incurred by the business.
5	Corporate body	A body incorporated under the Companies Act 1965 [Act 125].
6	Partnership	A business entity in which two or more people operate for the common goal of making profit. Each partner has total and unlimited personal liability of the debts incurred by the partnership
7	Society	An association, club or body that is registered under the Societies Act 1966 with a membership consisting of seven (7) or more members in addition to having specific purposes
8	Year commenced operations	The year during which the establishment began operating.
9	Individual Practice	Clinic practice run by one permanent doctor
10	Group Practice	Clinic practice run by more than one permanent doctor
11	General Practice Clinic	A clinic providing primary care services to the community. This service can be provided by a medical officer and/or a specialist in the field of family medicine
12	Specialist Clinic	A clinic with at least one resident specialist in a field besides family medicine that is accepted by the National Specialist Register of Malaysia.
13	Types of Klinik Kesihatan	Type 1:> 800 patient attendances per day<br>Type 2:500 - 800 patient attendances per day<br>Type 3:300 - 500 patient attendances per day<br>Type 4:150 - 300 patient attendances per day<br>Type 5:< 150 patient attendances per day<br>Type 6:< 50 patient attendances per day
14	Person coordinating this survey	Name of staff designated to liaise with the NHEWS Secretariat members on matters pertaining to NHEWS.

SECTION 2: FACILITIES (2010)

NO	DATA ELEMENT	DEFINITION
1	A triage system for assessing patients	A system that is dedicated to sorting patients into groups based on their need for or likely benefit from immediate medical treatment. It is the first clinic workstation encountered by patients upon arrival for medical review
2	Laboratory space	The work area within the clinic where laboratory support services are carried out. It usually consists of a "clean area" (for clerical, post analytical and validation work) and a dirty area (for pre analytical, analytical and sample buffering area).
3	Diagnostic/Imaging room	Designated room for x-ray device(s)
4	Ambulance services managed by your establishment	Emergency transport services that are run by staff that belong to the same management as the clinic.
5	Autoclaving facilities for sterilisation of medical equipment	Availability of the autoclave apparatus for the purpose of sterilisation of equipment used on patients. The apparatus may be in house or located away from the clinic premises

SECTION 3: HEALTHCARE ACTIVITIES & SERVICES (2010)

NO	DATA ELEMENT	DEFINITION
1	Office hours	From 8am to 5pm on weekdays and non-public holidays
2	Extended hours	The clinic is open and is providing services after office hours
3	After hours on call services	The clinic is closed however staff are on standby to provide medical services if required after office hours
4	Management of acute illnesses	Management of any illness characterized by signs and symptoms of rapid onset and short duration.
5	Management of chronic diseases	Management of any condition that has a protracted (usually ≥ 6 months) clinical course
6	Antenatal & postnatal care	Services rendered to patients during pregnancy and up to 42 days postpartum
7	Low risk deliveries	Delivery in a pregnancy that is anticipated to be problem free from antenatal to early intrapartum period. The assessment tool used to determine the status of the mother is the colour coding system. This tool indicates the status of the mother during that point of contact. When a mother has been continuously coded white throughout her pregnancy she is categorised as low risk and her labour can be conducted by a trained community health nurse (Jururawat Masyarakat)
8	Family planning	The conduct of educational and comprehensive medical activities which enable individuals to determine freely the number and spacing of their children and to select the means by which this may be achieved
9	Pap smear (cervical screening)	A simple smear method of examining stained exfoliative cells most commonly to detect cancers of the cervix
10	Clinical breast examinations	A physical examination of the breast performed by a health care provider to check for lumps or other changes.
11	Medical check-ups / examinations	A thorough physical examination that may include a variety of tests depending on the age, sex and health of the person
12	Occupational health	Health services concerned with the physical, mental and social well-being of an individual in relation to his/her working environment and with the adjustment of individuals to their work.
13	Smoking cessation programmes	Specifically designed programmes offered to patients to aid in their efforts to stop smoking tobacco products
14	Minor surgeries	Surgical procedures that can be conducted in a clinic setting without operating theatre facilities.
15	Attendance	An encounter between a patient and a healthcare professional in a clinic and includes all of the services provided during the encounter
16	Face to face consults	Encounter between a patient and a medical officer/ specialist that occurs in the clinic
17	Home visits	Encounter between a patient and a healthcare professional that occurs where the patient lives
18	Children attendances	Patient visits by persons below the age of 12 years

SECTION- 4,5,6

Copyright © National Healthcare Statistics Initiative. All rights reserved.
